# Effects of tight computerized glucose control on neurological outcome in severely brain injured patients: a multicenter sub-group analysis of the randomized-controlled open-label CGAO-REA study

**DOI:** 10.1186/s13054-014-0498-9

**Published:** 2014-09-05

**Authors:** Raphaël Cinotti, Carole Ichai, Jean-Christophe Orban, Pierre Kalfon, Fanny Feuillet, Antoine Roquilly, Bruno Riou, Yvonnick Blanloeil, Karim Asehnoune, Bertrand Rozec

**Affiliations:** Service d’Anesthésie-Réanimation. Hôpital Guillaume et René Laennec, Boulevard Jacques Monod, 44800 Saint-Herblain, CHU de Nantes, 44093 cedex France; Service de Réanimation médico-chirurgicale, Hôpital Saint-Roch, 5 rue Pierre Dévoluy, 06000 Nice cedex, CHU de Nice, France; INSERM U907 “Dysfonctionnements métaboliques et diabète : mécanismes et approches thérapeutiques”, Faculté de Médecine de Nice, 28 avenue de Valombrose, 06107 Nice, France; Service de Réanimation polyvalente, Hôpital Louis Pasteur, 4 rue Claude Bernard, 28630 Le Coudray, CH de Chartres, France; EA 4275 “Biostatistique, pharmaco-épidémiologie et mesures subjectives en santé”, Faculté de Pharmacie, Université de Nantes, 1 rue Gaston Veil, 44035 Nantes Cedex 1, France; Plateforme de Biométrie, Cellule de promotion de la recherche clinique, 1 rue Gaston Veil, 44035 Nantes Cedex 1, France; Service d’Anesthésie-Réanimation chirurgicale, Hôtel Dieu, 1 place Alexis Ricordeau, CHU Nantes, 44093 cedex France; Laboratoire UPRES EA 3826, Thérapeutiques cliniques et expérimentales des Infections, 1 rue Gaston Veil, 44035 Nantes Cedex 1, France; Service d’accueil des Urgences, CHU Pitié-Salpétrière, 47-83 boulevard de l’Hôpital, AP-HP, 75013 Paris, France; UMR INSERM 1166, IHU ICAN, Sorbonne Université, UMPC Univ Paris 6, 91 Buolivard de l’Hôpital, 75013 Paris, France; Institut du thorax, INSERM UMR1087 IRT, UN 8 quai Moncousu, BP 7072 44007 Nantes Cedex 1, France

## Abstract

**Introduction:**

Hyperglycemia is a marker of poor prognosis in severe brain injuries. There is currently little data regarding the effects of intensive insulin therapy (IIT) on neurological recovery.

**Methods:**

A sub-group analysis of the randomized-controlled CGAO-REA study (NCT01002482) in surgical intensive care units (ICU) of two university hospitals. Patients with severe brain injury, with an expected ICU length of stay ≥48 hours were included. Patients were randomized between a conventional glucose management group (blood glucose target between 5.5 and 9 mmol.L^−1^) and an IIT group (blood glucose target between 4.4 and 6 mmol.L^−1^). The primary outcome was the day-90 neurological outcome evaluated with the Glasgow outcome scale.

**Results:**

A total of 188 patients were included in this analysis. In total 98 (52%) patients were randomized in the control group and 90 (48%) in the IIT group. The mean Glasgow coma score at baseline was 7 (±4). Patients in the IIT group received more insulin (130 (68 to 251) IU versus 74 (13 to 165) IU in the control group, *P* = 0.01), had a significantly lower morning blood glucose level (5.9 (5.1 to 6.7) mmol.L^−1^ versus 6.5 (5.6 to 7.2) mmol.L^−1^, *P* <0.001) in the first 5 days after ICU admission. The IIT group experienced more episodes of hypoglycemia (*P* <0.0001). In the IIT group 24 (26.6%) patients had a favorable neurological outcome (good recovery or moderate disability) compared to 31 (31.6%) in the control group (*P* = 0.4). There were no differences in day-28 mortality. The occurrence of hypoglycemia did not influence the outcome.

**Conclusions:**

In this sub-group analysis of a large multicenter randomized trial, IIT did not appear to alter the day-90 neurological outcome or ICU morbidity in severe brain injured patients or ICU morbidity.

## Introduction

Numerous studies are available regarding blood glucose (BG) control in the intensive care unit (ICU) setting [1,2] and have led to the elaboration of international guidelines [3,4]. On the other hand, randomized controlled trials focusing on severely brain-injured patients, such as those with traumatic brain injury (TBI) or intra-cerebral hemorrhage (ICH), are scarce. Clinical studies are monocentric [5], frequently retrospective [6] and performed in small cohorts of patients [7]. Large randomized controlled trials did not evaluate specifically the impact of tight glucose control on neurological outcome in this specific ICU population [2]. Moreover, all of these studies have shown that this strategy increases the incidence of hypoglycemia that has been identified as an independent risk factor of mortality in the general-ICU setting [8] and has been advocated to increase cerebral glutamate and lactate/pyruvate ratio in TBI patients [9]. Therefore, balancing the potential beneficial effect of normalizing BG to the higher risk of hypoglycemia is a real matter of debate in brain-injured patients. Hyperglycemia has been clearly identified as a marker of poor outcome in TBI [10], cardiac arrest [11], ICH [12] and stroke [13]. If BG control appears mandatory in the neuro-ICU setting to prevent secondary brain damage and improve patient’s outcome [14], the appropriate BG target remains unclear.

Not only normoglycemia, but insulin itself has been reported to improve critically ill patients considering its metabolic and anti-inflammatory effects [15,16]. Experimental data suggest that insulin could increase astrocyte glucose uptake [17] and could play a role in cerebral glucose regulation in the cortex [18]. Finally, BG level after brain injury is more dependent on cerebral glucose utilization than BG level itself meaning that the appropriate glucose target to reach after brain insult remains unknown [19]. We conducted a sub-group analysis of a multicenter randomized-controlled open study (CGAO-REA study-NCT01002482) [20] regarding the effects of intensive insulin therapy (IIT) on neurological outcome in severely brain-injured patients.

## Material and methods

This study was a sub-group analysis of the non-blinded parallel-group randomized controlled CGAO-REA study (NCT01002482) [20] performed in two ICUs of French university hospitals (Nantes and Nice). Written informed consent was obtained before randomization, or delayed consent was obtained from each patient whenever neurological recovery was deemed appropriate or from a legal surrogate. The CGAO-REA study and ancillary studies were approved by the Ethics Committee of the teaching hospital of Tours, France.

### Inclusion criteria

As described in the CGAO-REA study, adults who required at least three days of ICU stay were eligible for this study. Patients in a moribund state at admission were not eligible.

All patients with a severe brain injury, with an expected ICU length of stay of at least three days at admission who were included in the CGAO-REA study, were eligible for this sub-group analysis; brain injuries included: TBI, aneurysmal subarachnoid hemorrhage (SAH), stroke, ICH without aneurysm, resuscitated cardiac arrest, brain tumor, cerebral abscess or central nervous system infection.

### Brain injury management

Brain-injured patients with a Glasgow coma score (GCS) ≤8 were intubated and were mechanically ventilated [21]. Patients were sedated with either midazolam (0.2 to 0.5 mg.kg^−1^.h^−1^) or propofol (1 to 5 mg.kg^−1^.h^−1^) and continuous infusion of fentanyl (2 to 5 μg.kg^−1^.h^−1^) or sufentanil (0.2 to 0.5 μg.kg^−1^.h^−1^). Management of patients was consistent with the guidelines of the Brain Trauma Foundation [22]. Subsequently, intra-cerebral pressure (ICP) monitoring was performed in patients with GCS ≤8 and with an abnormal brain computed tomography (CT) scan or whenever deemed appropriate by the attending intensivist, using either an intraparenchymental device or a ventriculostomy in the presence of hydrocephalus [23]. Cerebral perfusion pressure (CPP) was maintained in the range of 60 to 70 mmHg [21,22] with continuous infusion of norepinephrine when needed [21,22]. Since there is little evidence in the setting of neuro-vascular diseases regarding CPP thresholds, the same thresholds were applied in patients with ICP monitoring other than TBI, with respect to specific management, such as blood pressure targets following SAH or ICH [23]. To prevent secondary brain insults, the following standards of care were also applied: normoxia (PaO_2_ ≥ 80 mmHg), normocapnia (35 ≤ PCO_2_ ≤ 45 mmHg), body temperature between 36°C and 38°C and maintenance of a serum osmolality ranging between 280 and 320 mOsm.kg^−1^ [24].

Intracranial hypertensive episodes defined by an ICP ≥20 mmHg [22], were treated by boli of sedatives and a bolus of mannitol (0.5 g.kg^−1^) [25]. Mannitol was used in the setting of plasma osmolality ≤320 mosm.kg^−1^. In the case of refractory intracranial hypertension (ICP ≥20 mmHg for more than 15 minutes despite usual first-line treatment) [22], barbiturates (sodium thiopental) were added with an intravenous bolus of 2 to 3 mg.kg^−1^ followed by a continuous infusion of 2 to 3 mg.kg^−1^.h^−1^ [26]. Twenty-four hour therapeutic mild-hypothermia was a standard of care regarding resuscitated cardiac arrest [27] and was discussed in the setting of refractory ICP hypertension [28].

Sedation was stopped whenever the control of ICP was deemed appropriate.

### Blood glucose management

In the CGAO study, randomization was stratified according to the type of admission (scheduled surgical, emergency surgical, medical), diabetic status prior admission and conventional glucose control management in the ICU before the beginning of the study. BG management is described elsewhere [20]. Briefly, patients were included in the standard of care group (Control group) or the IIT group. In the IIT group, tight computerized BG control was performed with the assistance of the CGAO (Contrôle glycémique assisté par Ordinateur) software (LK^2^®, Saint-Avertin, France) set for targeting a BG range between 4.4 and 6 mmol.L^−1^. The CGAO software is an open-loop computer decision support system for BG control management that produces, at bedside, explicit recommendations regarding not only insulin titration (with an algorithm based on a proportional integral controller [29]), but also time for the next BG measurement and dose of intravenous glucose for correction of a possible hypoglycemia. The attending nurse could accept or decline the CGAO recommendations after each BG measurement, in the case of high doses of insulin administration which could lead to hypoglycemia. Nurses could ask the attending physician for assistance. In the control arm, BG management was based on current practice already used in the participating ICU before the beginning of the study and the targeted BG range was between 6 and 9 mmol.L^−1^ in Nantes and between 5.5 and 9 mmol.L^−1^ in Nice. All patients underwent an enteral nutrition protocol which included early initiation of enteral nutrition (day 1 after ICU admission), enteral intake target of 20 to 30 kCal.kg^−1^.day^−1^ [30] and the absence of residual gastric volume monitoring.

### Data collection

Patient characteristics and neurologic data at baseline were analyzed as well as BG level and mean doses of insulin within the first five days which was considered as the acute phase of brain-injury and intra-cerebral hypertension. Episodes of moderate (<3.3 mmol.L^−1^) and severe (<2.2 mmol.L^−1^) hypoglycemia were recorded.

### Outcome measures

The primary outcome was the neurologic outcome at day 90 following ICU admission and was assessed using the Glasgow outcome scale (GOS) via phone call blinded to treatment group [24]. Patients were dichotomized into good neurologic outcome defined by a good recovery and moderate disability (GOS 1 to 2) and poor neurologic outcome regarded as severe disability, vegetative state and death (GOS 3, 4, 5). Secondary outcomes were: neuro-surgical events during ICU stay, in-ICU death, neurologic outcome at day 28 following ICU admission, 28-day-ICU-free days and 28-day-ventilator-free days.

### Statistical analysis

Continuous data are expressed as mean ± standard deviation for parametric data and as median (25^th^ to 75th percentiles) for non-parametric data. Categorical data are expressed as number (%). A univariate analysis was performed regarding the primary and secondary outcomes between the control and the intervention groups. Parametric and non-parametric values were compared using the unpaired Student *t*-test and Mann-Whitney tests, respectively. Categorical values were compared with the chi^2^ test. We also performed a multivariate analysis regarding the risk factors of day 90 good neurological outcome [24]. Factors identified as potential prognosis factors for day 90 good neurological outcome by the univariate analysis with a cut-off *P* value at 0.2 were included in the logistic regression model and backward selection was applied. The calibration of the model was tested by a Hosmer-Lemeshow’s test. The final model was presented with a crude odds ratio (OR) and 95% confidence interval (CI). All *P* values were two-tailed and *P* values less than 0.05 were considered significant. Statistical analysis was performed with SAS statistical software (SAS 9.3 Institute, Cary, NC, USA).

## Results

A total of 496 patients from the ICUs of Nantes and Nice were included in the CGAO-REA study, and 188 of them (37%) were included in our sub-group analysis focused on brain-injured patients. Ninety four patients were included per center. Ninety (48%) patients were included in the IIT group and 98 (52%) in the control group. One hundred and seventy eight (95%) patients underwent mechanical ventilation for at least two days. The two groups were comparable except for GCS and body mass index (BMI), which were lower in the IIT group. In addition, more patients received ICP monitoring in the intervention group (n = 47, 52.2%) than in the control group (n = 34, 34.6%) (*P* = 0.01). Patient characteristics are provided in Table 1.

**Table 1:** **Characteristics of severe-brain injured patients**

	**Control group**	**IIT group**	***P*** **value**
	**Number = 98**	**Number = 90**	
Age	53 (15)	53 (16)	0.90
SAPS II	45 (15)	47 (17)	0.50
GCS on admission	7 (4)	6 (3)	0.02
Sex male/female	60(61)/38(39)	51(56)/39(44)	0.50
BMI	26 (5)	24 (4)	0.02
Diabetes mellitus	9 (9.2)	4 (4.4)	0.20
Laboratory glycemia on admission (mmol.l^−1^)	8.1 (6.8 to 9.8)	8.3 (6.8 to 9.6)	0.50
Monitoring of ICP	34 (35)	47 (52)	0.01^*^
Cause of brain injury, number(%)			0.50
Traumatic brain injury	19 (19)	22 (24)	
Aneurysmal subarachnoid hemorrhage	28 (29)	32 (36)	
Intra-cerebral hemorrhage	12 (12)	10 (11)	
Malignant stroke	11 (11)	5 (6)	
Resuscitated cardiac arrest	13 (13)	13 (14)	
Other	15 (15)	8 (9)	

Characteristics of patients included in the CGAO-REA study and suffering from a severe brain injury, in two neuro-intensive care units of two university hospitals. Continuous parametric data are expressed as mean (standard deviation) and non-parametric data as median (25^th^ to 75^th^ percentile) and categorical data as number (%). Continuous data were analyzed with Student’s t test. Categorical data were analyzed with χ^2^ test. BMI: body mass index (kg.m^−2^); GCS: Glasgow Coma Score; ICP: intracerebral pressure; IIT: intensive insulin therapy; SAPS II: Simplified Acute Physiology Score II.

Each morning laboratory BG was significantly lower in the IIT group (5.9 (5.1 to 6.7) mmol.L^−1^) than in the control group (6.5 (5.6 to 7.2) mmol.L^−1^) during the first five days of ICU hospitalization (*P* <0.001). In the IIT group, patients received significantly higher doses of insulin (130 (68 to 251) IU versus 74 (13 to 165) IU in the control group (*P* = 0.01)), within the first five days of ICU hospitalization. There were significantly more episodes of moderate hypoglycemia in the IIT group (n = 46, 51.1%) than in the control group (n = 19, 19.3%) (*P* <0.001). Six (6.67%) patients experienced an episode of severe hypoglycemia in the IIT group and four (4%) in the control group (*P* = 0.5). In-ICU glycemic events are summarized in Table 2.

**Table 2:** **In-ICU blood glucose events**

	**Control group**	**IIT group**	***P*** **value**
	**Number = 98**	**Number = 90**	
Median of the first five days morning laboratory blood glucose (mmol.l^−1^)	6.5 (5.6 to 7.2)	5.9 (5.1 to 6.7)	< 0.001^a^
Episodes of moderate hypoglycemia	19 (19.3)	46 (51.1)	< 0.001^b^
Episodes of severe hypoglycemia	4 (4)	6 (6.6)	0.50
Patients treated with insulin	81 (82.6)	87 (96.6)	0.002^b^
Total of insulin dose (IU) in the first five days	74 (13 to 165)	130 (68 to 251	0.01^a^

^a^Student’s t test; ^b^χ^2^ test. Moderate hypoglycemia was define as a blood glucose level <3.3 mmol.L^−1^. Severe hypoglycemia was defined as <2.2 mmol.L^−1^. Continuous data are expressed as median (25^th^ to 75th percentile) and categorical data as N (%). IIT: intensive insulin therapy.

### Outcomes

**Figure 1:**
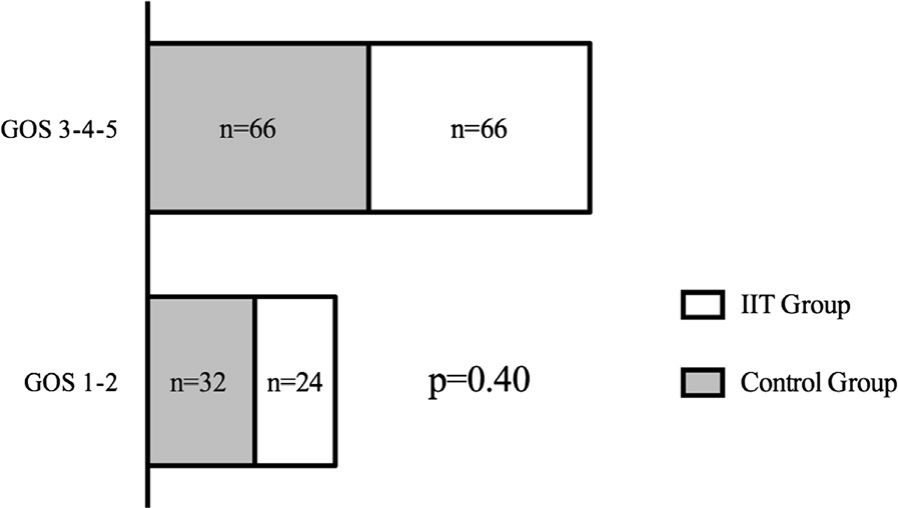
**Day 90 neurological outcome following ICU admission.** The figure represents the day-90 neurological outcome after ICU admission in severely brain-injured patients in the control group (blood glucose range between 5.5 and 9 mmol.L^−1^) and the intensive insulin therapy (IIT) group (blood glucose range between 4.4 and 6 mmol.L^−1^). Good neurological outcome is classified as a Glasgow outcome scale (GOS) score of 1 to 2 (good recovery, moderate disability). Poor neurological outcome is classified as a GOS score of 3, 4 or 5 (severe disability, vegetative state, death). χ^2^ test.

In the IIT group, 24 (26.6%) patients had a favorable neurological outcome (good recovery, moderate disability) compared to 32 (31.6%) in the control group (*P* = 0.40) (Figure 1). There were no significant differences regarding the day-28 neurological outcome, in-ICU death, the number of ventilation-free days and the number of ICU-free days (Table 3). There were no significant differences regarding in-ICU neuro-surgical events (Table 4). The occurrence of hypoglycemia did not significantly modify the day-90 neurological outcome (*P* = 0.70).

**Table 3:** **Outcome of severely brain-injured patients**

	**Control group**	**IIT group**	***P*** **value**
	**Number = 98**	**Number = 90**	
Ventilation-free days	8.5 (0 to 22)	9 (0 to 20)	0.40
ICU-free days	8.5 (0 to 20)	8 (0 to 20)	0.50
Day-28 mortality	28 (28.6)	26 (28.9)	0.90
Day-28 good neurologic outcome	31 (31.6)	24 (26.6)	0.40

Crude outcome of severely brain-injured patients included in a sub-group analysis of the CGAO-REA study. Ventilation and ICU-free days are expressed between ICU admission and Day-28. Good neurologic outcome was defined as a good recovery or moderate disability (Glasgow outcome scale score 1 to 2) [24]. Poor neurologic outcome was defined as severe disability or vegetative state or death (Glasgow outcome scale score 3, 4, 5) [24]. Continuous data are expressed as median (25^th^ to 75^th^ percentile) and categorical data as number (%). Continuous data were analyzed with Student’s t test. Categorical data were analyzed with χ^2^ test. IIT: intensive insulin therapy.

**Table 4:** **Neuro-surgical events in the ICU in severely brain-injured patients**

	**Control group**	**IIT group**	***P*** **value**
	**Number = 98**	**Number = 90**	
Patients presenting at least one episode of ICP ≥25 mmHg during ICP monitoring	17 (17.3)	23 (25.5)	0.20
Administration of mannitol during ICU	19 (19.4)	28 (31.1)	0.06^*^
Barbiturates use during ICU	9 (9.2)	14 (15.6)	0.20

Specific neuro-surgical events in the ICU in a sub-group analysis of severely brain-injured patients in two university hospitals of the CGAO-REA study. Categorical data are expressed as number (%) and analyzed with χ^2^ test. ICP: intra-cerebral pressure; IIT: intensive insulin therapy.

### Prognosis factors of favorable day-90 neurological outcome

On univariate analysis, the prognosis factors of favorable neurological outcome were: other cause of brain injury than brain trauma, the absence of decompressive craniectomy, the absence of nosocomial pneumonia during ICU hospitalization, lower blood glucose level at ICU admission, administration during ICU of anti-epileptic drugs, a higher administration of insulin dose in the first five days after ICU admission and a higher number of ventilation-free days. In the multivariate analysis, all causes of brain injury except trauma, administration of anti-epileptic drugs and a higher number of ventilation-free days were independent and significant prognosis factors of favorable outcome (Table 5).

**Table 5:** **Exploratory multivariate analysis of risk factors of day-90 favorable neurological outcome**

	**Adjusted OR**	**CI**_**95%**_ **(OR)**	***P*** **value**
Cause of brain injury			0.004
Traumatic brain injury	1		
Neuro-vascular cause (SAH, ICH, malignant stroke)	1.27	(0.46 to 3.54)	
Resuscitated cardiac arrest	3.99	(1.02 to 15.61)	
Other etiologies (brain tumor, central nervous system infection, cerebral vascularitis)	7.92	(2.10 to 29.91)	
Anti-epileptic drugs	2.99	(1.10 to 8.16)	0.03
Number of ventilation-free days	1.11	(1.07 to 1.16)	<0.001

Risk factors of day-90 favorable neurological outcome defined as good recovery and moderate disability. Hosmer and Lemeshow goodness-of-fit test *P* = 0.94. CI_95%_: 95% confidence interval; ICH: intracerebral hemorrhage; OR: odds ratio; SAH: subarachnoid hemorrhage.

## Discussion

Our study shows that tight computerized BG control had no significant effect on neurological recovery at day-90 following ICU admission in the sub-group of brain-injured patients extracted from the multi-center randomized-controlled CGAO-REA trial. The incidence of moderate hypoglycemia was higher in the IIT group but this did not significantly modify neurological outcome.

Acute neurological injuries bear significant mortality and are one of the major causes of severe disability among young healthy individuals in western countries and incur substantial health-care costs [23,31]. Hyperglycemia is a well-recognized secondary brain insult and is a marker of poor outcome in every brain-injured patient, leading to increased brain damage [10]. BG control is, therefore, mandatory and has proved efficacious in improving neurological outcome in stroke [14]. However, such results have not been clearly evaluated in ICU patients with neurologic diseases and high ICP. Few studies have been performed in the neuro-ICU setting. In a single center randomized study IIT did not result in any neurologic improvement in a critically ill neurologic population [32]. In a large series of 178 SAH patients, the authors pointed out that an elevated BG level (>7.8 mmol.L^−1^) was associated with a poorer outcome, but most patients displayed a mild to moderate form of SAH [33]. Also, the authors used a conventional BG control. In a large retrospective analysis of a single-center cohort of various neurologic injuries, Graffignano *et al*. [6] reported that IIT was associated with more episodes of hypoglycemia, an increased in-hospital length of stay and a higher mortality. In this study, the severity of neurologic injury and the occurrence of intracranial hypertension were unknown [6]. It is, therefore, difficult to draw conclusions from such studies about the appropriate BG level to target in the neuro-ICU setting.

Yang *et al*. [5] performed a randomized controlled study in severe traumatic brain-injured patients. Interestingly, the IIT group with a 4.4 to 6.1 mmol.L^−1^ target exhibited a lower infections rate, lower ICU length of stay and a better six-month neurologic outcome evaluated with the GOS. The main drawback in this study is that the control group received insulin only when patients had a BG level >11.1 mmol.L^−1^, which is not a standard of care [2], and no data were available regarding the management of insulin. In experimental rat models of TBI, the provision of glucose has been reported to improve cerebral metabolism and decrease neuronal injury [34], and insulin resulted in an increased astrocyte glucose uptake [17], suggesting that a large intake of glucose combined with a high insulin infusion after acute neurological injury could prevent secondary brain damages. To the best of our knowledge, we provide the first results obtained in a multi-center study regarding BG management in severely brain-injured patients and we failed to demonstrate any significant difference in the neurological outcome between patients treated with IIT or conventional BG control.

It is noteworthy that a slight lowering in BG levels in our intervention group was associated with a higher incidence of moderate hypoglycemia. Several studies suggest that hypoglycemia is an independent mortality factor in the ICU [2], which was not observed in the CGAO-REA study [20]. Data regarding hypoglycemia and neurologic diseases are conflicting. In a study including 14 consecutive TBI patients undergoing IIT, Vespa *et al*. [35] demonstrated an increased cerebral glutamate and lactate/pyruvate ratio and low cerebral glucose assessed with local microdialysis. There was, however, no relationship between serum glucose rates and global rates of glycolysis, evaluated with positron emission tomography suggesting the possible lack of connection between serum and cerebral glucose. In this study, it is also unknown whether these cerebral stress markers have long-term clinical consequences [35]. Moreover, recent data suggest a neuroprotective role of lactate during hypoglycemia, as the brain shifts lactate utilization over glucose, when both substrates are available [36]. In an experimental model, Suh *et al*. [37] have recently demonstrated that BG reperfusion is responsible for neuronal cell death after hypoglycemia which was not responsible *per se* for neuronal apoptosis. Finally, in our study, as well as other ICU studies regarding BG control, several relevant hypoglycemia severity markers [38], such as duration of hypoglycemic episodes and hypoglycemic-related neurological signs, are not available, mostly because of sedation or previous neurological impairment. Put together, all these issues make it difficult to ascertain the potential neurological consequences of hypoglycemia in severely brain-injured patients.

Risk factors associated with favorable neurological outcome were resuscitated cardiac arrest or miscellaneous causes of brain injury compared to TBI, the administration of anti-epileptic drugs during ICU stay and the number of ventilation-free days (Table 5). Studies in resuscitated cardiac arrest usually focus on in-hospital mortality and neurological status at hospital discharge [39], which makes it difficult to compare to TBI. This result must be cautiously interpreted and remains purely exploratory. Administration of anti-epileptic drugs in the early course of TBI [40] or SAH [23] is mandatory to prevent secondary seizure following brain injury. However, there is little evidence on whether early seizure prophylaxis improves long-term outcome.

There is growing evidence that respiratory complications frequently occur in brain-injured patients [41] and could impact the outcome [42]. It is therefore interesting that a higher number of ventilation-free days was independently associated with a better outcome in our study. An evidence-based bundle is able to reduce the duration of mechanical ventilation in severely brain-injured-patients [43] but there is not yet enough data to assess the impact of such a strategy on the long-term neurological outcome.

Our study has several limitations. This is a sub-group analysis of a large multi-center study and our results remain purely exploratory. In the initial study [20], there was no stratification on the type or severity of neurologic injury. It is, therefore, possible to retrieve an imbalance of patient’s severity. Nonetheless, hyperglycemia is a source of secondary brain damage and a marker of poor prognosis in all types of brain injury and one can expect that IIT could have a potential effect on neurological recovery. Finally, IIT could serve a beneficial role in neurologic recovery; this effect could remain undetected since patients in the IIT group had a greater neurologic severity at baseline (lower GCS in this group). In our centers, barbiturates and osmotherapy were commonly used in the most severe patients. To the best of our knowledge, IIT is not advocated to induce ICP hypertension. We believe that the use of barbiturates and osmotherapy probably reflect the severity of brain injury.

Despite significant differences, BG levels were very close in both groups without any real clinical relevance, blunting a possible clinical impact of close BG control on neurological recovery in severely brain-injured patients. In the same way, the absence of hypoglycemia-related neurological worse outcome must be cautiously interpreted because most of the hypoglycemic episodes were moderate. Lastly, the severity of hypoglycemia was only defined by its biological threshold without considering either the duration of these episodes or the usual clinical signs of hypoglycemia.

## Conclusions

Tight BG control did not result in an improved neurological outcome in severely brain-injured patients but significantly increased the rate of moderate episodes of hypoglycemia. Since the most appropriate BG threshold in this specific ICU-population remains unknown, a moderate BG management goal between 5.5 and 9 mmol.L^−1^ seems preferable.

## Key messages

- Intensive insulin therapy does not affect outcome in severely brain-injured patients.
- Intensive insulin therapy does not affect ICU morbidity in severely brain-injured patients.
- Hypoglycemia is not a marker of poor neurological outcome in severely brain-injured patients.

## Abbreviations

BG: blood glucose
CI: confidence interval
CPP: cerebral perfusion pressure
GCS: Glasgow Coma Score
GOS: Glasgow Outcome Scale
ICH: intra-cerebral hemorrhage
ICP: intracranial pressure
IIT: intensive insulin therapy
OR: odds ratio
SAH: subarachnoid hemorrhage
TBI: traumatic brain injury
